# Involvement of the Transient Receptor Channels in Preclinical Models of Musculoskeletal Pain

**DOI:** 10.2174/1570159X21666230908094159

**Published:** 2023-09-08

**Authors:** Sabrina Qader Kudsi, Fernanda Tibolla Viero, Leonardo Gomes Pereira, Gabriela Trevisan

**Affiliations:** 1 Programa de Pós-Graduação em Farmacologia, Universidade Federal de Santa Maria (UFSM), Avenida Roraima, 97105-900 Santa Maria (RS), Brazil

**Keywords:** Gastrocnemius, tibial, masseter, muscle afferents, CFA, Carrageenan

## Abstract

**Background:**

Musculoskeletal pain is a condition that affects bones, muscles, and tendons and is present in various diseases and/or clinical conditions. This type of pain represents a growing problem with enormous socioeconomic impacts, highlighting the importance of developing treatments tailored to the patient's needs. TRP is a large family of non-selective cation channels involved in pain perception. Vanilloid (TRPV1 and TRPV4), ankyrin (TRPA1), and melastatin (TRPM8) are involved in physiological functions, including nociception, mediation of neuropeptide release, heat/cold sensing, and mechanical sensation.

**Objective:**

In this context, we provide an updated view of the most studied preclinical models of muscle hyperalgesia and the role of transient receptor potential (TRP) in these models.

**Methods:**

This review describes preclinical models of muscle hyperalgesia induced by intramuscular administration of algogenic substances and/or induction of muscle damage by physical exercise in the masseter, gastrocnemius, and tibial muscles.

**Results:**

The participation of TRPV1, TRPA1, and TRPV4 in different models of musculoskeletal pain was evaluated using pharmacological and genetic tools. All the studies detected the antinociceptive effect of respective antagonists or reduced nociception in knockout mice.

**Conclusion:**

Hence, TRPV1, TRPV4, and TRPA1 blockers could potentially be utilized in the future for inducing analgesia in muscle hypersensitivity pathologies.

## 1 INTRODUCTION

Musculoskeletal pain stands as the most prevalent form of discomfort, impacting approximately 13.5% to 47% of the overall population. Consequently, this type of pain compromises the quality of life for individuals affected by this condition [1-3]. It is characterized by pathological conditions that affect ligaments, joints, and bones, often generating painful or localized sensations that can radiate to regions of the body beyond the directly affected area. Also, this form of pain is reported as the most prevalent pain among individuals enduring chronic pain [4].

According to the International Classification of Diseases 11 (ICD-11), developed by the International Association for the Study of Pain (IASP) in partnership with representatives of the World Health Organization (WHO), chronic musculoskeletal pain can be categorized as either primary or secondary. Primary chronic musculoskeletal pain cannot be specifically attributed to any pathology or damage, as occurs in fibromyalgia [4]. Secondary chronic musculoskeletal pain arises due to some underlying disease for which the painful sensation could be considered one of the symptoms, as in traumatic muscle injury [4, 5].

Skeletal muscle injuries can be caused by a range of events, including direct trauma (such as muscle lacerations, contusions, or strains) and indirect causes (such as ischemia, infection, or neurological dysfunction) [6]. Besides, 90% of the cases of muscle injuries are due to strains [6-9]. After skeletal muscle injury, a general mechanism of damage and repair occurs and is divided into 4 sequential steps: degeneration, inflammation, regeneration, and fibrosis [7, 10-12]. The processes of muscle degeneration and inflammation occur in the first days after the injury when muscle fibers are ruptured and hematoma forms. Although most skeletal muscle injuries heal without the formation of fibrous scar tissue, fibroblast proliferation, in some cases, can be excessive, resulting in scar tissue formation. The vascularization process is very important, as it helps in muscle regeneration and recovery of the morphological structure of the muscle [7, 10-12].

During the inflammatory phase, several inflammatory mediators can be released or produced at the injury site, such as prostaglandins, bradykinin, cytokines, chemokines, and nitric oxide [13-16]. The muscle injury is followed by increased Ca^2+^ influx into the muscle cells, resulting in muscle passive tension and myofibrillar disruption. The production of reactive oxygen species (ROS) and cytokines promoting the activation of transcription factors such as nuclear factor-kappa B (NF-κB), mitogen-activated protein kinase (MAPK), and nuclear factor erythroid 2-related factor 2 (*Nrf2*) subsequently trigger secondary inflammatory responses. In addition, some inflammatory cells can release ROS and cytokines such as neutrophils, phagocytic, and macrophages [17-19]. These substances, when in contact with the nociceptive terminals, can cause sensitization of nociceptors (peripheral and central), promoting symptoms in patients such as allodynia and hyperalgesia [14, 20, 21].

Several receptors can be expressed in skeletal muscle, inflammatory cells, and muscle nociceptive afferents, such as transient receptor channels (TRP) [22-24]. According to Kudsi *et al.* (2022) [24], there was a bias in human skeletal muscle tissue in TRPV1 transcription levels greater than TRPA1, TRPV4, and TRPM8. Furthermore, the expression of receptors such as acid receptors (ASICs) and purinergic receptors (P2X) in models of muscle hyperalgesia has been extensively studied [25, 26]. Furthermore, the expression of receptors such as acid receptors (ASICs) and purinergic receptors (P2X) in models of muscle hyperalgesia has been extensively studied [25, 26]. Muscle fatigue causes muscle acidosis, and proton production mainly activates ASIC3 receptors [25], while high ATP concentration activates P2X_3_ or P2X_7_ receptors [26, 27].

In clinical practice, non-pharmacological and pharmacological treatments for musculoskeletal pain are still ineffective, which makes it necessary to search for new effective and safe therapeutic alternatives that reduce pain, improve inflammation, and help in the functional recovery of the injured muscle [28]. The most used non-pharmacological treatments are cryotherapy, laser therapy, therapeutic ultrasound, and the use of protocols such as PRICE (Protection, Rest, Ice, Compression, and Elevation) [9, 29-31]. On the other hand, non-steroidal anti-inflammatory drugs (NSAIDs), non- opioid analgesics (paracetamol), opioids (morphine, codeine, and tramadol), and muscle relaxants (carisoprodol, cyclobenzaprine, orphenadrine, tizanidine, and baclofen) stand out [32]. Although pharmacological treatments are relatively effective in the treatment of muscle pain and inflammation, their prolonged use can lead to deficits in muscle regeneration due to reduced proliferation and differentiation of satellite cells, with consequent formation of thinner and weaker muscle fibers, in addition to the accumulation of connective tissue [33]. Thus, it is necessary to look for new therapeutic targets for more effective treatments in musculoskeletal pain.

## 2 TRP AND MUSCULOSKELETAL PAIN

The TRP channels are part of a large, non-selective, cation-permeable family of membrane proteins [34]. These ion channels are involved in various biological processes such as chemosensation [35], mechanosensation [36], thermosensation [35], and nociception [37] and can also be related to various diseases or pathological conditions [24, 38, 39]. Besides, TRP channels share features comprising six transmembrane proteins and an intracellular amino and carboxy domain [35, 40].

The superfamily of TRP channels in mammals consists of 28 members [41, 42] subdivided into six subgroups according to their amino acid sequence homology: TRPC (Canonical 1-7), TRPV (Vanilloid 1-6), TRPM (Melastatin 1-8), TRPP (Polycystin 1-3), TRPML (Mucolipin 1-3), and TRPA (ankyrin 1) [41, 42]. Among these six subfamilies, the most studied receptors related to muscular hyperalgesia are TRPV1, TRPA1, and TRPV4 [24, 43].

TRPV1 is a non-selective cation-permeable channel that can be activated by noxious heat (above 43°C), pH (acid, < 6.0), and various natural products such as capsaicin (found in chili peppers), and resiniferatoxin (present in *Euphorbia resinifera*) [44, 45]. The TRPV1 channel can be expressed preferentially in sensory neurons of the dorsal root ganglion (DRG) and trigeminal ganglion (TG), especially in peptidergic C-fibres [44-46]. TRPV1 can be expressed in non-neuronal tissues such as keratinocytes, macrophages, and neutrophils [24, 45]. Also, TRPV1 can be found in skeletal muscle and participates in muscle physiology, being relevant for muscle contraction [22-24]. In rats, TRPV1 is functionally expressed in skeletal muscle, preferably at the sarcoplasmic reticulum (SR) membrane in the proximity of SERCA1 pumps [22]. TRPV1 acts as a reticular Ca^2+^-leak channel, and TRPV1 mutations are associated with muscle disorders such as malignant hyperthermia (MH) and exertional heat stroke (EHS) [23].

Besides, this receptor has been studied in different models of inflammatory and neuropathic pain using antagonists or genetic-deleted mice [47, 48]. Capsaicin, a potent TRPV1 agonist, can be used in the clinical setting for the control of neuropathic pain conditions, where it defunctionalizes the nociceptors, causing long-lasting analgesia [49]. Moreover, different studies investigated the expression of TRPV1 in muscular nociceptive afferents and muscular tissue to explore the participation of this receptor in muscular hyperalgesia [48-61]. The subcutaneous injection of capsaicin into the skin causes burning pain by the activation of unmyelinated nociceptors in humans [62]. Besides, this TRPV1 agonist is also capable of activating muscular nociceptors, causing pain [63]. Then, the first study that evaluated muscular afferents and TRPV1 channels used unmyelinated muscle afferents (group IV) of the gastrocnemius-soleus muscle of rats. The authors showed that these fibers are sensitive to acid (pH 6.0), capsaicin, and ATP, with some overlapping responses. Also, the TRPV1, ASIC, and P2X_3_ receptors were found in retrogradely labeled DRG cells innervating the gastrocnemius-soleus muscle, especially DRG neurons with small sizes. Then, capsaicin can activate mechanosensitive group IV muscle fibers to cause nociceptor activation [64-66]. Also, TRPV1 was found in afferents from the colon and quadriceps muscle (a large percentage) and in skin afferents from mice's hind paws. The muscle afferents expressing TRPV1 showed a large range of size distribution, whereas visceral and cutaneous had small soma sizes [24, 67].

Another study described the localization of TRPV1-positive nociceptive fibers that innervate the gastrocnemius and erector spinae muscles in the DRG. The TRPV1 channels were expressed in gastrocnemius (49%) and erector spinae (40%) muscle afferents. The majority of these fibers also expressed calcitonin gene-related peptide (CGRP), with some of them showing P2X_3_ expression. Besides, the injection of acid into gastrocnemius and erector spinae muscle caused the expression of phosphor extracellular signal-regulated kinase (pERK) with close contact with TRPV1-positive fibers in the spinal cord. This suggests the potential involvement of TRPV1-positive afferents in muscular pain, with a predominant peptidergic fiber composition observed in rats [68]. In a different study, it was described the electrophysiological characteristics of TRPV1-positive muscle afferents in DRG in mice (innervating the gastrocnemius muscle), which were isolectin B4 (IB4)-negative. The authors showed that these afferents were all responsive to acid. Thus, TRPV1 is a sensor for muscular hyperalgesia caused by acid [69].

Besides, the injection of capsaicin into masseter muscle causes pain in humans [70, 71]. In this view, a study showed that masseter afferent neurons with cell bodies into TG respond to capsaicin, ATP, and acid challenge. These ion channels, TRPV1, ASIC, and P2X_3_, were also expressed in masseter afferents in TG [72]. Besides, the distribution of TRPV1, TRPV2, and P2X_3_ was also found in the trigeminal ganglion retrogradely labeled from masseter muscle, and 19% of them co-expressed TRPV1 and CGRP in rats [73]. A different research group demonstrated that in mice, innervation of the masseter muscle within the trigeminal ganglion (TG) revealed the presence of TRPV1-expressing small-sized neurons that were positive for calcitonin gene-related peptide (CGRP), along with afferents expressing IB-4+/TRPA1- or IB-4+/TRPA1+ [74].

TRPA1 is a non-selective channel permeable to cations such as calcium, sodium, and potassium, although it has a much higher calcium preference than the other TRP channels [35]. The TRPA1 channel can be activated by chemical irritants such as cinnamaldehyde (found in cinnamon), allyl isothiocyanate (AITC, present in mustard oil), and also by oxidative stress products such as hydrogen peroxide (H_2_O_2_) and lipid peroxidation products such as 4-hydroxynonenal (4-HNE) [75, 76]. TRPA1 can be expressed in the dorsal root ganglion, trigeminal ganglion, and visceral neurons [16, 77]. Meanwhile, on the non-neuronal level, it is expressed in keratinocytes, dendritic cells, neutrophils, astrocytes, and Schwann cells [78]. The TRPA1 is also expressed in human primary myoblasts, where it leads to calcium influx when activated by TRPA1 agonists. This channel is also involved in muscle repair (cell migration and myoblast fusion), but the mRNA levels for *Trpa1* are reduced after myoblast differentiation. Thus, this receptor could be relevant in the early stages of muscle repair and in the activation of quiescent satellite cells *in vitro* [79]. Moreover, this channel has been extensively studied in models of neuropathic, cancer, and inflammatory pain [24]. However, until now no TRPA1 antagonist or agonist is used in the clinical setting for pain control [35]. Besides, related to TRPA1 expression in muscular afferents or participation in muscular pain we have only some studies [48, 49, 54, 80], showing the relevance to the study of this channel in this type of pain.

Another TRP channel studied for pain induction is the TRPV4, this receptor is also a non-selective cation-permeable channel. The TRPV4 can be activated by various factors such as mechanical stimuli, hyposmolarity, heat (above 34°C), and H_2_O_2_ [81, 82]. Besides, it is activated by metabolites produced by arachidonic acid (AA) such as epoxyeicosatrienoic acid (EET) [83, 84]. At the neuronal level, TRPV4 can be expressed in TG sensory neurons [43, 85], DRG [43, 86]. At the non-neuronal level, TRPV4 can be found in chondrocytes [87, 88], keratinocytes [89], odontoblasts [90, 91], astrocytes [92, 93], and microglial cells [94, 95]. This TRP channel is expressed in TG neurons and other cells, including temporomandibular joint fibroblast-like synoviocytes of rodents [82, 96]. The TRPV4 channel is also functionally expressed in skeletal muscle according to Pritschow *et al.* (2011) [97]. TRPV4 may be functionally expressed in the skeletal muscle of mice, and TRPV4 activation modulates the influx of Ca^2+^ at rest and reduces muscle fatigue in mice [97]. The TRPV4 has been investigated for the induction of inflammatory and neuropathic pain [82]. Besides, TRPV4 is also expressed in muscle nociceptive afferents [98] and could be involved in muscular hyperalgesia as described in this review.

**Fig. (1):**
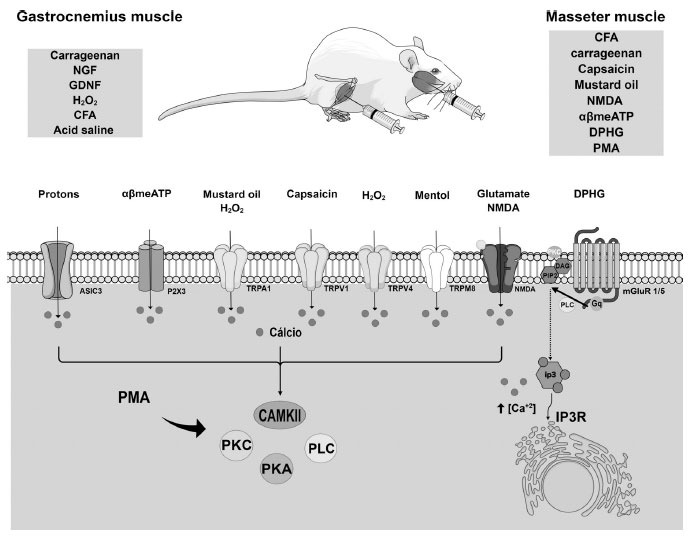
Models of muscular hyperalgesia in the gastrocnemius and masseter muscle in rodents and the participation of transient receptor potential receptors (TRPs). The different models of muscle hyperalgesia in the masseter muscle used algogenic substances, such as complete Freund's adjuvant (CFA), carrageenan, capsaicin (TRPV1 agonist), mustard oil (TRPA1 agonist), N-methyl-D-aspartate (NMDA, TRPA1 activator), alphabetameATP (αβmeATP, TRPA1 activator), dihydroxyphenylglycine (DPHG, mGlu1/5 agonist), phorbol 12-myristate 13-acetate (PMA, an activator of PKC), capsaicin (TRPV1 agonist), or mustard oil (TRPA1 and TRPV4 agonist). The models of muscle hyperalgesia in the gastrocnemius muscle used nociceptive substances such as CFA, carrageenan, nerve growth factor (NGF promotes proliferation, degranulation, and release of inflammatory mediators from immune cells), glial cell-derived neurotrophic factor (GDNF promotes proliferation, degranulation, and release of inflammatory mediators from immune cells), H_2_O_2_ (TRPA1 and TRPV4 agonist), and acid saline (promotes acidosis that activates TRPs and ASIC3), ATP (activates P2X_3_). The indirect or direct release of calcium by activating these receptors leads to the activation of protein kinases such as PKA, PKC, PLC, and CAMKII. In this sense, these mechanisms have the capability to trigger multiple pain and inflammation signaling pathways.

Thus, in this review, we will explore the studies that described the participation of TRPV1, TRPA1, and TRPV4 in models of muscular nociception in rodents. Besides, we included only articles involving the study of muscular models of pain by direct injury to a muscle (masseter, gastrocnemius, or tibial) (Fig. **1**). Thus, other models used to study fibromyalgia or different musculoskeletal conditions were not described in this study, but recent reviews described these findings [24, 99].

## 3 ROLE OF TRP CHANNELS IN OROFACIAL MUSCULAR NOCICEPTION DETECTED IN THE MASSETER MUSCLE

Orofacial pain is one of the most frequent types of chronic pain, with a prevalence of 5-33% worldwide being a frequent social and medical challenge [100-102]. This type of pain is detected in various conditions, including headaches, temporomandibular joint disorders, trigeminal neuropathy, and dental pain. In this sense, the most common diagnosis of chronic orofacial pain is temporomandibular disorders (TMD) (55.3%) [103].

TMD involves the masticatory muscles and/or temporomandibular joints, often triggering orofacial pain accompanied by noise and joint mobility restriction [104, 105]. TMD patients may have alterations in central nociceptive processing, which is thought to be triggered by a peripheral source, possibly from the masticatory muscles [106], as nociceptive inputs from inflammatory muscles are a potent generator of central sensitization [107]. Therefore, by inducing inflammation with algogenic substances in the masseter muscle or even in the TMJ joint, trigeminal ganglia, muscle, and afferents, it is possible to evaluate the mechanisms that evolve orofacial pain. These studies could be helpful to promote better treatment for this type of pain [108].

While TMJ has multifactorial etiologies, in a subset of patients with TMJ, pain is caused by masticatory muscle injury or joint inflammation. Different algogenic agents can be administered in the masseter muscle to mimic orofacial muscle pain, including complete Freund’s adjuvant (CFA), carrageenan, N-methyl-D-aspartate (NMDA, an NMDA activator), alphabetameATP (αβmeATP, a P2X_3_ activator), dihydroxyphenylglycine (DPHG, mGlu1/5 agonist), phorbol 12-myristate 13-acetate (PMA, an activator of PKC), capsaicin (a TRPV1 agonist), or mustard oil (TRPA1 activator) (Fig. **1**) [50-57, 80]. The CFA or carrageenan injection into the masseter muscle has been used as a model of chronic inflammatory orofacial pain in rodents [109-111]. CFA contains heat-killed *Mycobacterium tuberculosis,* and it is a standard compound used to induce inflammation in several animal models due to mast cell activation, leukocyte infiltration, and the production of free radicals and cytokines in circulation [112]. Carrageenan is a sulfated polysaccharide extracted mainly from an alga and is a widely used reagent known for its ability to induce acute inflammation and edema [112]. Then, these are common models of inflammatory pain in the literature used to study the mechanisms involved in peripheral and central sensitization of pain in the orofacial region [52-54, 80, 113].

Furthermore, Chung and colleagues (2016) [114] demonstrated the possible changes in protein expression in TG in a model of muscle inflammation in rats. The authors performed an RNA-seq assay to characterize transcriptome profiles of genes related to nociception. They detected an increased expression of different TRP channels mRNAs (*Trpv1, Trpa1, Trpm8, and Trpm3*), but TRPV4 expression was reduced, and other TRP expressions were not altered (*Trpv3*, *Trpv2*, *Trpm2*, *Trpc7*, *Trpc6*, *Trpc3*, and *Trpc4*) [114].

Regarding rodent models of muscle hyperalgesia, although the hippocampus is not the most significant area in the brain for pain processing, this structure has been studied in various pain conditions. In addition to the TG expression, the TRPV1 receptor is found in the hippocampus and can directly mediate synaptic plasticity. Findings of upregulation of TRPV1 in the hippocampus but not in other brain regions after muscle inflammation suggest a role for hippocampal TRPV1 in TMD [115]. Thus, to test whether bilateral nocifensive behavioral response after unilateral masseter muscle inflammation caused by CFA injection is due to TRPV1 activation in the hippocampal, a specific TRPV1 antagonist 5-iodoresiniferatoxin was injected into the CA1 region of the structure [50]. This study observed that the unilateral injection of CFA into the masseter muscle caused bilateral mechanical allodynia, which was partially attenuated by 5-iodoresiniferatoxin. However, the unilateral injection of CFA into masseter muscle up-regulates TRPV1 expression in ipsilateral and does not affect TRPV1 expression level in contralateral TG. This remains the sole article that has evaluated the role of hippocampal TRPV1 following masseter inflammation. However, it would also be interesting to evaluate the role of TRPV1 in the TG since upregulation induced by CFA has been demonstrated in this structure.

Furthermore, Wang and colleagues (2017) [51] also demonstrated the role of TRPV1 in CFA-mediated masseter inflammation in mice using pharmacological and genetic tools. Muscle inflammation increased spontaneous nociception (measured by mouse grimace scale and face-wiping behavior), which was reduced in *Trpv1^−/−^* mice and after TRPV1 antagonist injection (AMG9810, into the masseter muscle). A reduction in bite force was also detected after CFA or carrageenan injection into the masseter muscle. This nociceptive parameter was slightly reduced in *Trpv1^−/−^* mice or after AMG9810 administration after CFA injection, but for the carrageenan model, *Trpv1^−/−^* mice also showed nociception. Another critical area for orofacial nociception is the trigeminal spinal subnucleus caudalis (Vc), one of three nuclei in the sensory trigeminal nerve pathway. This area, jointly with TG and the upper cervical spinal cord, acts in neuroplastic changes in the neuronal networks and is an important mechanism underlying orofacial pain. Thus, it was performed the ablation of TRPV1-expressing neurons in Vc with resiniferatoxin (RTX, a potent TRPV1 agonist); this procedure reduced MGS and bite force triggered by CFA-induced masseter inflammation. In a different protocol, clozapine-N-oxide (CNO) was injected in the masseter of TRPV1-Cre or TRPV1-hM4Di to cause chemogenetic silencing of TRPV1-lineage neurons. TRPV1-hM4Di injected with CNO presented reduced masseter-associated nociception to capsaicin or CFA injection. Finally, NK1-expressing neurons were ablated by SP-Sap (neurotoxin saporin conjugated to substance P) to Vc, and the ablation of NK1-expressing second-order neurons reduced the MGS and bite force nociceptive behaviors. These results suggest that TRPV1 and NK1-expressing second-order neurons contribute to spontaneous and bite-evoked pain during masseter inflammation and muscle pain. Therefore, blocking nociceptive terminals TRPV1 might be a potential therapeutic to treat spontaneous muscle pain [51].

After this study, Wang *et al.* (2018) [52] investigated whether TRPA1 also contributes to spontaneous pain or bite-evoked pain in mice and if TRPV1 and TRPA1 may produce an addictive effect in CFA-induced masseter inflammation. First, the authors administered AP18, a specific antagonist of TRPA1, into masseter muscle after CFA injection, and this compound reduced the MGS score. A similar effect was detected when *Trpv1^−/−^* mice were injected with CFA, then TRPA1 is relevant for MGS score increase after CFA injection in the masseter muscle. However, AP18 treatment of *Trpv1^−/−^* mice did not attenuate face-wiping behaviors. Moreover, during muscle inflammation, stress is increased, such as H_2_O_2_, which is a TRPA1 agonist [52, 70]. Thus, catalase enzyme was injected into masseter muscle after CFA injection and significantly reduced MGS scores during masseter inflammation. Also, they postulated that scavenging putative ligands of TRPV1 might produce a similar analgesic effect. Thus, authors evaluated the participation of oxidized linoleic acid metabolites (OLAMs), such as 9(S)-HODE or 13(S)-HODE, suggested to be endogenous ligands of TRPV1. The authors also demonstrated that simultaneous antagonism of TRPA1 by AP18 and TRPV1 by AMG9810 in masseter muscle resulted in the inhibition of both MGS and face-wiping behaviors, without additive antinociceptive effect [52]. Besides, administration of AP18 or AMG9810 to masseter muscle-induced conditioned place preference. The extent of conditioned place preference following simultaneous administration of AP18 and AMG9810 was more significant than that induced by the injection of individual antagonists [52].

In contrast, bite-evoked pain was not reduced by the inhibition of TRPA1 alone or in combination with TRPV1. This study was a pioneer in demonstrating that TRPV1 and TRPA1 mediate spontaneous pain in an additive form, probably by endogenous ligand activation generated in inflamed muscle [52].

Orofacial pain is more commonly related to women than males [116]. Besides, clinical studies have revealed that females show more pain-related sensations than males induced by TRPV1 agonists [117, 118]. In this sense, Bai *et al.* (2018) [53] demonstrated that CFA-induced orofacial nociception increases the levels of *Trpv1* mRNA and protein in the TG of female rats following CFA injection into masseter muscle with no alteration for male rats. Thus, the role of testosterone in TRPV1 expression was assessed in ovariectomized animals that received testosterone replacement for 7 days following CFA exposure mice. Testosterone seems to inhibit the upregulation of TRPV1 after CFA injection in ovariectomized rats. However, tamoxifen (a competitive estrogen receptor antagonist and partial agonist) treatment in ovariectomized female rats did not prevent increased TRPV1 expression after CFA injection to the masseter muscle. In this view, the authors demonstrated that testosterone might be involved in maintaining the TRPV1 increased expression in this model of orofacial pain [53]. Besides, it would also be interesting to study the effectiveness of the TRPV1 or TRPA1 antagonists in ovariectomized and ovariectomized rats.

In a different study, it was described the participation of TRPV1 and TRPA1 receptors in craniofacial muscle nociception using intramuscular injection of capsaicin (a TRPV1 agonist) or mustard oil (a TRPA1 agonist) in masseter muscle [54]. The authors also showed in this study that TRPV1 and TRPA1 are expressed in masseter afferents innervating the TG and are likely to be colocalized in masseter afferents. Subsequently, capsaicin injection to the masseter muscle caused nociception. These nociceptive behaviors were reduced by the pretreatment with capsazepine (a TRPV1 antagonist) injection to the ipsilateral masseter muscle. A selective TRPV1 antagonist (AMG9810), when pre-injected to the ipsilateral masseter muscle, reduced the capsaicin-induced mechanical hypersensitivity. Mustard oil (a TRPA1 agonist) injection to the masseter muscle also caused nociception. Besides, the local ipsilateral pre-injection of AP18 or HC-030031 produced an antinociceptive effect, showing that TRPA1 selective antagonists may cause the reduction in channel activation by MO in the masseter muscle. Thus, the blockage of local TRPV1 or TRPA1 receptors could be studied as a future mechanism for the control of muscle nociception [54].

Additionally, glutamate release has been described as a proposed pathway to induce masseter muscle hypersensitivity. In this view, activation of glutamate receptors such as N-methyl-D-aspartate receptor (NMDAR) could induce calcium influx and subsequent activation of protein kinases such as protein kinase A (PKA), protein kinase C (PKC) and calcium calmodulin-dependent protein kinase II (CaMKII), leading to TRPV1 sensitization. Thus, Lee *et al.* (2012) [55] injected NMDA into the masseter muscle causing mechanical nociception, which was blocked by local pretreatment with an NMDAR antagonist (AP5), a CAMKII inhibitor (KN93), a PKC inhibitor (GF109203X) and a PKA inhibitor (KT5720). Pretreatment with a TRPV1 antagonist (AMG9810, 100 nmol/site) in the masseter muscle also reduced NMDA-induced nociception but did not block the mechanical hypersensitivity caused by a TRPA1 agonist (mustard oil). TRPV1 is also co-expressed with the NR1 subunit of NMDARs in 32% of masseter afferents of TG. Besides using co-immunoprecipitation techniques, the authors also demonstrated the possibility of protein-protein complexes between TRPV1 and NR1/NR2 subunits of NMDAR in TG neurons. In TG neurons, NMDA causes potentiation of capsaicin-mediated calcium influx, showing the functional interaction between these receptors. Finally, the authors demonstrated that NMDA activation caused the phosphorylation of TRPV1 (serine residues) expressed in cultured TG neurons. Also, the increased phosphorylation of TRPV1 was reduced by the pre-treatment of cultured TG with a CAMKII inhibitor (KN93) and a PKC inhibitor (GF109203X), with no effect for a PKA inhibitor (KT5720). Thus, NMDA and TRPV1 receptors functionally interact in the TG neurons, promoting masseter muscle nociception, and the NMDA activation leads to TRPV1 phosphorylation at specific residues by PKC and CAMKII pathways [55].

Furthermore, the TRPV1 receptor could be sensitized by pro-inflammatory mediators, including adenosine triphosphate (ATP), which may be released into muscle during injury or inflammation [56]. P2X_3_, an ionotropic purinergic receptor expressed in sensory neurons, is involved in masseter nociception following agonist intramuscular injection (αβmeATP) in rats. The pre-treatment reduced the nociception with a TRPV1 (AMG9810, 10 and 100 nmol/site) or a P2X_3_ (A-317491) antagonist into the masseter ipsilateral side. This non-selective cation channel (P2X_3_) is co-expressed with TRPV1 in 7.2% of masseter afferents of TG neurons. Also, in a similar way proposed to the NMDA model, P2X_3_ activation leads to TRPV1 serine residue phosphorylation in TG culture, and P2X_3_ activation sensitizes the TRPV1 in TG culture when calcium influx is analyzed. However, the authors did not evaluate the effect of specific kinases in TRPV1 phosphorylation mediated by P2X_3_ (Saloman *et al.*, 2013) [56]. Then, TRPV1 and other ionotropic receptors (NMDA or P2X_3_) may interact to cause muscle nociception after masseter injury or inflammation.

Subsequently, the same research group proposed another mechanism involving glutamate activation of metabotropic receptor mGlu1/5 to induce TRPV1 sensitization [114]. DPHG (dihydroxyphenylglycine, a mGlu1/5 receptor agonist), when injected into the masseter muscle of rats, provoked nociception. TRPV1 antagonist pre-treatment (AMG9810) reduced DPHG and PMA (PKC activator) induced masseter hypersensitivity, but this compound did not decrease muscle nociception caused by phosphorylated (PKA activator). The disruption of A-kinase anchoring protein (AKAP) and TRPV1 interactions by using a membrane-permeable decoy peptide (736-745-TAT) may reduce TRPV1 phosphorylation, then in this model, 736-745-TAT pre-treatment reduced DPHG-induced masseter nociception. The TRPV1 phosphorylation (serine residues, as S800) mediated by DPHG was reduced in TG culture neurons by a PKC inhibitor; also, in electrophysiology studies, TRPV1 was sensitized by DPHG by a PKC-dependent pathway (Chung *et al.*, 2015) [114]. Thus, TRPV1 may function as a danger signal integrator in the masseter muscle following injury, inducing pain and sensitization, especially glutamate activation of NMDA, mGlu1/5, and P_2_X_3_ receptors.

Moreover, in a distinct study, the same research group investigated the possibility of TRPA1 sensitization by NMDAR and P_2_X_3_ receptors. In this sense, Asgar *et al.* (2015) [80] described that αβmeATP injection in the masseter-induced nociception (using lightly anesthetized rats), which was blocked by the pre-treatment with TRPA1 antagonists (AP18 or HC-030031). Similarly, AP18 or HC-030031 reduced NMDA-induced nociception in the masseter muscle. The authors also demonstrated that CFA injection in the masseter induced mechanical hypersensitivity, spontaneous muscle pain responses, and significant up-regulation of TRPA1 expression in TG. These nociceptive behaviors were significantly reversed by post-treatment of the muscle with AP18 (Asgar *et al.*, 2015) [80]. Thus, TRPA1 mediates acute muscle mechanical hypersensitivity induced by ATP and glutamate. These data suggested that TRPA1 may serve as a downstream target of pro-nociceptive ion channels, such as P_2_X_3_ and NMDA receptors in masseter afferents.

The participation of TRPV1 and TRPA1 in different models of orofacial muscular pain was evaluated using pharmacological and genetic tools (Table **1**). Then, in conclusion, all the studies detected the antinociceptive effect of respective antagonists or reduced nociception in knockout mice. The upregulation of TRPV1 in the hippocampus but not in other brain regions after muscle inflammation suggests a role for hippocampal TRPV1 in TMD. In TG, NMDA and TRPV1 receptors functionally interact to promote masseter muscle nociception. Furthermore, TRPV1 seems to mediate masseter pain, especially after activation of NMDA, mGlu1/5, and P2X_3_ receptors. TRPA1 mediates acute mechanical nociception induced by ATP and glutamate that may be a downstream target of pro-nociceptive ion channels, such as P2X_3_ and NMDA receptors in masseter afferents. Furthermore, the simultaneous pharmacological antagonism of TRPA1 and TRPV1 reduced spontaneous nociception without additive effect.

Considering the sexual dimorphism presented in orofacial pain, testosterone seems to inhibit the upregulation of TRPV1 in TG. However, most studies used only males for the experiments. Thus, it is still poorly known about the possible different downstream mechanisms between sexes (Table **1**).

## 4 ROLE OF TRP CHANNELS MUSCULAR NOCICEPTION DETECTED IN THE GASTROCNEMIUS AND TIBIAL MUSCLES

Muscular hyperalgesia is described as an unpleasant sensation or tenderness, principally after unaccustomed strenuous exercise, such as delayed-onset muscle soreness (DOMS) [119]. Mechanisms underlying referred muscle pain involve a variety of endogenous mediators generated during injury or inflammation that sensitize nociceptive afferent fibers and contribute to the development of muscular pain [25, 33, 120]. Considering that TRP receptors are primarily expressed on peripheral and central terminals of nociceptors, it is possible to suggest that activation of these receptors is essential to sensitization induced by final inflammatory mediators in primary afferent fibers [25, 121]. As described above, for the masseter muscle, the inflammation and induction of nociception in gastrocnemius or tibial muscles could be done using different protocols.

Initially, the role of TRP channels was studied using the carrageenan injection into the gastrocnemius muscle, which produced muscular mechanical hyperalgesia (only in the Randall-Selitto test) accompanied by inflammatory cell migration to muscular tissue. However, there was no increase in TRPV1 or TRPV2 expression in DRG (L4-L6) samples after inflammation of the gastrocnemius muscle or alteration in DRG retrograde labeled neurons innervating the muscle. TRPV1 channel is mainly found in muscle sensory neurons retrogradely labeled small to medium-size, and TRPV2 was found in medium to large-sized neurons. The intraperitoneal or intramuscular injection of capsazepine (a TRPV1 antagonist) did not reduce mechanical hyperalgesia caused by carrageenan injection, but ruthenium red (a TRP antagonist) partially reduced muscular nociception. Eccentric exercise of the gastrocnemius muscle caused mechanical hyperalgesia but did not induce inflammatory cell migration to muscle tissue or altered expression of TRPV1 or TRPV2 channels. Also, the intraperitoneal injection of ruthenium red reduced mechanical hyperalgesia caused by eccentric exercise, which was not reduced by capsazepine injection. Besides, the intramuscular injection of both TRP antagonists reduced muscular nociception. The authors also showed that acid-sensing ion channels (ASICs) receptors are involved in muscular nociception in both models. The eccentric exercise protocol is also considered a model of delayed-onset muscle soreness (DOMS), and in this model, TRPV1 and ASICs have a relevant involvement [58]. This was the first paper using models of muscular pain to study the role of TRP channels. Initially, it seems that TRPV1 is not relevant in this model of inflammatory muscle pain caused by carrageenan injection. However, the authors did not use *Trpv1^−/−^* mice or explore other doses of the TRPV1 antagonists. Although the non-selective TRP antagonist (ruthenium red) reduced muscular nociception in both models, thus nociception could be maintained by another TRP channel to be studied.

Moreover, another study also indicated the involvement of TRPV1 and TRPV4 in muscular hypersensitivity caused by neurotrophin injection into the lateral gastrocnemius muscle and in a model of DOMS. Ota and colleagues demonstrated that nerve growth factor (NGF) injection did not induce muscular mechanical hyperalgesia in *Trpv1^−/−^* mice but caused nociception in WT and *Trpv4^−/−^* mice. However, the intramuscular injection of glial cell-derived neurotrophic factor (GDNF) did not cause mechanical hyperalgesia in both *Trpv4^−/−^* and *Trpv1^−/−^* mice. Also, in this study, authors induced lengthening contraction (LC) mainly of the lateral gastrocnemius muscle by electrical stimulation of the tibial nerve as a model of DOMS. The authors showed that TRPV1 and TRPV4 are involved in muscular mechanical hyperalgesia because *Trpv1^−/−^* or *Trpv4^−/−^* mice were protected from nociception induced by this model. Also, the LC model increased NGF expression (lateral gastrocnemius muscle) in *Trpv1^−/−^* and *Trpv4^−/−^*, but it only enhanced GDNF levels in *Trpv4^−/−^* mice. Besides, the injection of a TRPV4 antagonist (HC-067047) before and after LC induction did not alter GDNF expression. In this view, TRPV1 and TRPV4 are involved in DOMS detected in the lateral gastrocnemius muscle, but TRPV1 and TRPV4 channels are involved downstream to neurotrophins up-regulation, and TRPV4 may also be involved upstream to these pathways to induce muscular pain [98].

Similarly, Murase and colleagues (2012) [59] also demonstrated that GDNF injection to the gastrocnemius muscle induced a persistent decrease in the mechanical muscle withdrawal threshold. Capsazepine (a TRPV1 antagonist) intramuscular treatment did not induce an antinociceptive effect. In contrast, amiloride treatment (a non-selective ASICs inhibitor) was able to reverse the GDNF-induced mechanical hyperalgesia. Besides, intramuscular injection of GDNF could sensitize muscular Aδ-fiber afferents, but not C-fibers, when mechanical stimulation was done in the periphery. Also, there was no difference between heat-sensitive and insensitive C-fibers in the mechanical response threshold. The results might indicate that GDNF acts on sensory neurons expressing ASICs with larger diameters (Aδ myelinated afferent fibers) [59]. In this view, the role of neurotrophins in muscular pain should be further evaluated to detect TRP receptors' participation in muscular nociceptor sensitization.

In addition, a model of gastrocnemius muscle inflammatory pain caused by carrageenan injection was used to test the role of TRPV1 channels in this type of inflammatory pain. Carrageenan injection caused mechanical allodynia, similar in *Trpv1^+/+^* and *Trpv1^−/−^* mice. However, the heat hyperalgesia caused by carrageenan injection into the gastrocnemius muscle was absent in *Trpv1^−/−^* mice. Then, the reexpression of TRPV1 in the muscle and skin of *Trpv1^−/−^* mice restored heat hyperalgesia after carrageenan injection. These results suggest that TRPV1 mediated nociceptor sensitization after muscle inflammation [61].

Furthermore, Chen *et al.* (2014) [61] demonstrated the involvement of TRPV1 in a model of muscular hyperalgesia induced by acid saline injection into the gastrocnemius muscle. In this study, authors administered acid saline in two different time points, *Trpv1^−/−^* mice or intramuscular injection of a TRPV1 antagonist (capsazepine) reduced long-lasting muscular hyperalgesia. A similar antinociceptive effect was detected for *Asic3^−/−^* mice and the co-treatment with an ASIC3 antagonist (APETx2). Additionally, it remains unknown how the injection of acidic saline activates the TRPV1 channel to preserve chronic hyperalgesia [61].

Moreover, Fang *et al.* (2020) [49] showed that CFA injection to the anterior tibialis muscle caused nociception and increased the spontaneous activity of muscular sensory afferents in rats. The reduction in weight-bearing caused by CFA injection was prevented by the application of topical irritants on the skin near the inflamed muscle, including capsaicin (a TRPV1 agonist), mustard oil (a TRPA1 agonist) or peppermint oil (a TRPM8 agonist). The denervation of skin over the muscle or L4 dorsal rhizotomy did not reduce nociception in this model. The study demonstrated that activating TRP channels evoked by capsaicin, mustard oil, and peppermint oil reduced muscle nociception *via* activating cutaneous nociceptors and reducing altered activity in these neurons after muscle inflammation [49]. Capsaicin application is a pharmacological treatment for neuropathic pain and other chronic pathologies, and it could be used for local muscular pain, such as muscle sprains and strains [122, 123].

In another study, the role of TRPA1 was investigated. Sugiyama and collaborators (2017) [48] examined the role of this channel in a model of postoperative pain caused by skin and deep muscle incisions in rats and H_2_O_2_-induced muscle nociception. The treatment with a TRPA1 antagonist (HC-030031, intraperitoneal injection before or after incision) reduced guarding behavior after skin and deep muscle incision (plantar flexor digitorum brevis muscle). However, the injection of HC-030031 could not reduce mechanical and heat hypersensitivity caused by skin and deep muscle incisions. Also, the ROS imaging *in vivo* shows an increase in the luminescent probe after skin or muscle incision (gastrocnemius muscle). This event was reduced after the injection of catalase (implying that the ROS increase was partly attributable to an increase in H_2_O_2_) in the muscle. The production of H_2_O_2_ was also increased in the gastrocnemius muscle and skin after the incision. Besides, the injection of H_2_O_2_ in the gastrocnemius muscle caused nociception, which was reduced by HC-030031 injection into the muscle tissue. However, in rats, H_2_O_2_ subcutaneous injection into the tissue overlying the gastrocnemius muscle did not induce nociception [48].

The induction of muscle inflammation or eccentric exercise caused nociception in the studies described above, and the role of TRPV1, TRPV4, and TRPA1 was evaluated. However, all the studies used male rats or mice, and it would be interesting to evaluate the nociceptive parameters in female rodents. Moreover, while TRPV1 has been extensively studied, there is a need for additional research to investigate other TRP channels that are also expressed in muscular tissue, such as those found in the gastrocnemius or tibial muscles, as well as within muscular nociceptive afferents. The expression of TRP channels was not evaluated in all the studies; therefore, it would be premature to conclude that injury or inflammation of the gastrocnemius muscle caused altered expression of TRP channels. Ultimately, only a single article presented the potential of diminishing nociception through topical application of TRP agonists, suggesting it could serve as an alternative strategy to reduce muscular pain (Table **2**).

## 5 OTHER MODELS OF MUSCULOSKELETAL PAIN

When assessing muscle hyperalgesia, additional models involve injury induced by intense exercise, including treadmill-based workouts [124, 125], alongside various other methodologies [10].

Initially, Abdelhamid *et al.* (2013) [125] studied the participation of TRPV1 in muscular hyperalgesia caused by forced swimming in female mice (Swiss Webster, 20-25 g). Nociception caused by forced swimming (at 26°C) involved the corticotropin-releasing factor 2 (CRF2) receptors. whereas CRF2 or TRPV1 receptors did not exhibit involvement. The nociception was evaluated using the grip forced test, and mice were pre-treated with CRF1 antagonist (NBI-35965, intraperitoneal injection), CRF2 antagonist (Astressin 2B), TRPV1 antagonist (SB-366791), or were centrally or peripherally desensitized with the TRPV1 agonist (resiniferatoxin, intrathecal administration). Consequently, muscular nociception detected in this model is not dependent on the TRPV1 channel [125]. However, the authors did not evaluate the expression of TRPV1 receptors or other doses of the antagonist, but these data showed no involvement of TRPV1 in acute stress-induced muscular nociception.

Moreover, Retamoso *et al.* (2016) [124] used a treadmill-based exercise model in male rats and investigated the TRPV1 protein content. The authors studied the mechanisms involved in DOMS and then used a protocol where rats were exposed to downhill running exercise on a treadmill and after a grip force test and mechanical allodynia were evaluated. After the induction of this model, rats showed nociception, increased protein expression of TRPV1 (12 hours after exercise), and oxidative stress markers (carbonyl content and xanthine oxidase activity) in the gastrocnemius muscle [124]. In this study, it was observed that eccentric exercise led to the development of mechanical allodynia and an elevation in TRPV1 levels within the injured muscle.

## CONCLUSION

The participation of TRPs in models of musculoskeletal pain was evaluated using pharmacological and genetic approaches. All the studies demonstrated the antinociceptive effect of antagonists in knockout mice. Models of muscle hyperalgesia induced by algogenic substances, trauma, and physical exercise cause tissue acidosis and mechanical, cold, and heat hyperalgesia. In addition, most animals are under anesthesia for evaluation of nociceptive and behavioral tests, causing a large bias. In addition, to date, there are no defined studies on the innervation of muscle fibers in different regions of the muscle. While the majority of articles mention extensive innervation within the fascia, there are no studies yet substantiating this claim. Basbaum *et al.* (2009) [126] already described in physiological studies the population of nociceptive fibers that innervate the skin and their participation in pain, pruritus, and temperature, which is a gap regarding nociceptive muscle fibers. Furthermore, we emphasize that novel studies should also explore the role of different TRPs in musculoskeletal pain and evaluate the antinociceptive effect of TRP antagonists on female rodents. TRPA1, TRPV1, and TRPV4 could be the target of promising pharmacological strategies for musculoskeletal pain models, mainly spontaneous nociception and mechanical allodynia. On the other hand, TRPM8 had only one study using menthol, a compound widely used in several formulations for treating musculoskeletal pain. It may be a potential target for further studies involving muscle hyperalgesia.

## Figures and Tables

**Table 1:** Models of muscular hyperalgesia in the masseter muscle in rodents and the participation of transient receptor potential receptors (TRPs).

Rodent Type	Pain Muscle Model	Measure Pain	Receptor Expression	Treatment	Route, Dose, Time	References
Sprague Dawley rats (Male, 250-350 g).	CFA (50%, unilaterally) into masseter muscle.	Mechanical head withdrawal threshold for masseter muscle stimulation (Electronic von Frey anesthesiometer).	TRPV1 (RT-PCR in TG).	TRPV1 antagonist (5-iodoresiniferatoxin).	5-iodoresiniferatoxin (0.1 and 0.5 nmol/site, hippocampus CA1 region, bilaterally).	Simonic-kocijan *et al.*, 2013 [50]
*Trpv1*^-/-^, *Trpv1*-Cre mice crossed with R26-hM4Di, and *Trpv1*^+/+^ mice (Male, 8-16-week-old).	CFA (50%, unilaterally or bilaterally); carrageenan (3%, bilaterally); capsaicin (3%, bilaterally) into the masseter muscle.	Spontaneous nociception (MGS, face-wiping behaviour) and bite force.	TRPV1 (IHC, Vc and TG masseter afferents).	TRPV1 antagonist (AMG9810); TRPV1 agonist (RTX).	AMG9810 (200 nmol/site, masseter muscle, bilaterally); RTX (50 ng/site, Vc, bilaterally).	Wang *et al.*, 2017 [51]
*Trpv1*^-/-^, *Trpa1*^-/-^, *Trpv1*^+/+^, and *Trpa1*^+/+^ mice (Male, 8-16-week-old).	CFA (50%, unilaterally or bilaterally) into masseter muscle.	Spontaneous nociception (MGS, face-wiping behaviour, and CPP) and bite force.	-	TRPV1 antagonist (AMG9810) and TRPA1 antagonist (AP18).	AP18 (100 nmol, 300 nmol and 800 nmol/site, bilaterally); AMG 9810 (200 nmol/site, bilaterally).	Wang *et al.*, 2018 [52]
Sprague Dawley rats (Female and male 250-350 g, 8-week-old). Orchidectomized (male) or ovariectomized (female). Testosterone replacement (male) or tamoxifen injection (female).	CFA (50%, unilaterally) into masseter muscle.	Mechanical threshold for masseter muscle stimulation (von Frey filaments).	TRPV1 (WB, RT-PCR in TG).	-	-	Bai *et al.*, 2018 [53]
Sprague Dawley rats (Male, 250-350 g).	Capsaicin (a TRPV1 agonist, 0.001-0.1%/ site, unilaterally) and mustard oil (a TRPA1 agonist, 1-10%/site, unilaterally) injection into masseter muscle.	Hind paw shaking responses and mechanical threshold for the hind paw response to masseter muscle stimulation (electronic von Frey anesthesiometer).	TRPV1 and TRPA1 (IHC, TG masseter afferents).	TRPV1 antagonists (capsazepine and AMG9810) and TRPA1 antagonists (AP18 and HC030031).	Capsazepine (200 nmol/site, unilaterally); AMG9810 (100 nmol and 1 µmol/site, unilaterally); AP18 (1 µmol/site, unilaterally); HC030031 (1 and 10 µmol/site, unilaterally) injection in the masseter muscle.	Ro *et al.*, 2009 [54]
Sprague Dawley rats (Male, 150-350 g).	NMDA (10 nmol/ site, unilaterally) and mustard oil (a TRPA1 agonist, 20%/site, unilaterally) injection into masseter muscle.	Mechanical threshold for the hind paw response to masseter muscle stimulation (electronic von Frey anesthesiometer).	TRPV1 (IHC, TG masseter afferents).	TRPV1 antagonist (AMG9810).	AMG9810 (10 and 100 nmol/site, unilaterally) injection into masseter muscle.	Lee *et al.*, 2012 [55]
Sprague Dawley rats (Male, 100-350 g).	αβmeATP (250, 500, and 750 μg/ site, unilaterally) injection to the masseter muscle.	Mechanical threshold for the hind paw response to masseter muscle stimulation (electronic von Frey anesthesiometer).	TRPV1 (IHC, TG masseter afferents; WB, TG culture).	TRPV1 antagonist (AMG9810).	AMG9810 (5, 10 and 100 nmol/site, unilaterally) injection into masseter muscle.	Saloman *et al.*, 2013 [56]
Sprague Dawley rats (Male, 150-350 g)	DPHG (1 μmol/ site; unilaterally) and PMA (300 nmol/site; unilaterally) injection to the masseter muscle.	Mechanical threshold for the hind paw response to masseter muscle stimulation (electronic von Frey anesthesiometer).	TRPV1 (WB, TG culture).	TRPV1 antagonist (AMG9810).	AMG9810 (1, 10 and 100 nmol/site, unilaterally) injection into masseter muscle.	Chung *et al.*, 2015 [57]
Sprague Dawley rats (Male, 250-350 g)	NMDA (10 μmol, unilaterally); αβmeATP (750 μg, unilaterally); CFA (50%, unilaterally) into masseter muscle.	Spontaneous nociception (RGS); mechanical threshold for the hind paw response (electronic von Frey anesthesiometer) and mechanical hypersensitivity to the masseter muscle. stimulation (von Frey filaments).	TRPA1 (WB, RT-PCR in TG).	TRPA1 antagonists (AP18 and HC030031).	AP18 (10 nmol/site - 2 μmol/site, unilaterally); HC-030031 (50 nmol/site, unilaterally).	Asgar *et al.*, 2015 [80]

**Note**: *Models of muscular hyperalgesia in the masseter muscle in rodents and the participation of transient receptor potential receptors (TRPs). Complete Freund adjuvant (CFA); Trigeminal ganglion (TG); Transient Receptor Potential Cation Channel Subfamily A (Ankyrin) Member 1 (TRPA1); Transient Receptor Potential Cation Channel Subfamily V (Vanilloid) Member 1 (TRPV1); Mouse grimace scale (MGS); Immunohistochemistry (IHC); Western Blot (WB), Real-time reverse transcriptase-polymerase chain reaction (RT-PCR); trigeminal nucleus caudalis (Vc); Resiniferatoxin (RTX); conditioned place preference (CPP); α,β-methylene ATP (αβmeATP); dihydroxyphenylglycine (DPHG); Phorbol 12-myristate 13-acetate (PMA); Rat grimace scale (RGS).

**Table 2:** Models of muscular hyperalgesia in the gastrocnemius and tibial muscles and transient receptor potential receptors (TRPs) participation in rodents.

Rodent Type	Pain Muscle Model	Measure Pain	Receptor Expression	Treatment	Dose, Time, Route	References
Sprague Dawley rats (Male, 250 g).	Carrageenan injection into gastrocnemius muscle (50 µL, 4%), exercise protocol of eccentric gastrocnemius muscle contraction by electrical stimulation of the tibial nerve.	Mechanical withdrawal threshold of the deep tissues (Randall-Selitto test, skin over the gastrocnemius muscle); mechanical withdrawal threshold (von Frey test, skin over the gastrocnemius muscle).	TRPV1 and TRPV2 (RT-qPCR), IHC (DRG, retrogradely labelled gastrocnemius sensory neurons).	TRPV1 (capsazepine) and TRP non-selective (ruthenium red) antagonists.	Capsazepine (30 mg/kg, intraperitoneal or 100 or 1000 pmol/50 µL, intramuscular), ruthenium red (3 mg/kg, intraperitoneal or 100 or 1000 pmol/50 µL, intramuscular).	Fujii *et al.*, 2008 [58]
*Trpv1*^-/-,^*Trpv4*^-/-,^*Trpv1*^+/+^ and *Trpv4*^+/+^ mice (Male, adult).	Lengthening contraction (LC) of lateral gastrocnemius muscle by electrical stimulation of the tibial nerve, murine NGF-2.5 S (0.8 µM, 5 µl, i.m.) or recombinant murine GDNF (0.03 µM, 5 µl) injection into lateral gastrocnemius muscle.	Mechanical withdrawal threshold of the deep tissues (Randall-Selitto test, skin over the lateral gastrocnemius muscle); mechanical withdrawal threshold (von Frey test, skin over the lateral gastrocnemius muscle).	-	TRPV4 antagonist (HC-067047).	HC-067047 (10 or 100 mg/kg, 10 µL) in gastrocnemius muscle.	Ota *et al.*, 2013 [98]
Sprague Dawley rats (Male, 290-330 g).	GDNF injection in lateral gastrocnemius muscle (0.015, 0.03 μM, 20 µL).	Mechanical withdrawal threshold of the deep tissues (Randall-Selitto test, skin over the lateral gastrocnemius muscle).	-	TRPV1 antagonist (capsazepine).	Capsazepine (50 μM) was injected into the gastrocnemius muscle.	Murase *et al.*, 2014 [59]
*Trpv1*^-/-^ and *Trpv1*^+/+^ mice.	Carrageenan injection into gastrocnemius muscle (20 µL, 3%)	Mechanical withdrawal threshold (von Frey test, hind paw), heat withdrawal latency (Hargreaves test, hind paw).	-	Recombinant herpesviruses for re-expression TRPV1 channels (HSV-GFP-TRPV1).	Two injections of 10 µL of viruses into the gastrocnemius muscle.	Walder *et al.*, 2012 [60]
*Trpv1*^-/-^ and *Trpv1*^+/+^ mice (Male, adult, 8- to 12-wk-old).	Received 2 injections, spaced 1-5 days apart, of acid saline pH 4.0 (20 μL) into gastrocnemius muscle.	Mechanical withdrawal threshold (von Frey test, hind paw).	-	TRPV1 antagonist (capsazepine).	Capsazepine (1 nmol) into the gastrocnemius muscle.	Chen *et al.*, 2014 [61]
Sprague-Dawley rats (Female, 180-220g).	CFA injection to the tibialis anterior muscle (100 µL)	Weight-bearing measurement.	-	Capsaicin (a TRPV1 agonist), peppermint oil (a TRPM8 agonist), and mustard oil (a TRPA1 agonist).	Capsaicin (0.3%, 0.5 mL), peppermint oil (80%, 0.5 mL), and mustard oil (50%, 0.5 mL) was applied to the hindlimb.	Fang *et al.*, 2020 [49]
Sprague-Dawley rats (Male, 200-320 g).	Skin and deep tissue incision (plantar flexor digitorum brevis muscle and gastrocnemius muscle), H_2_O_2_ (100 mM, 0,6 mL) injection in the gastrocnemius muscle or subcutaneous tissue overlying gastrocnemius muscle.	Guarding behavior (pain score, hind paw), mechanical withdrawal threshold (von Frey test, hind paw), heat withdrawal latency (Hargreaves test, hind paw), and nociceptive time (hind paw).	-	TRPA1 antagonist (HC-030031).	75, 150, and 300 mg/kg (intraperitoneal); 100 mM (0.3 mL, into the gastrocnemius muscle).	Sugiyama *et al.*, 2017 [48]

**Note**: *Models of muscular hyperalgesia in the gastrocnemius and tibial muscles and the participation of transient receptor potential receptors (TRPs) in rodents. Transient Receptor Potential Cation Channel Subfamily V (Vanilloid) Member 1 (TRPV1); Transient Receptor Potential Cation Channel Subfamily V (Vanilloid) Member 2 (TRPV2); Transient Receptor Potential Cation Channel Subfamily V (Vanilloid) Member 4 (TRPV4); Transient receptor potential cation channel subfamily M (melastatin) member 8 TRPM8; Immunohistochemistry (IHC); Real-time reverse transcriptase-polymerase chain reaction (RT-qPCR); Dorsal root ganglia (DRG); Lengthening contraction (LC); Nerve growth factor (NGF); Glial cell line-derived neurotrophic factor (GDNF); Herpes simplex virus (HSV); Green fluorescent protein (GFP); Complete Freund's adjuvant (CFA); Hydrogen peroxide (H_2_O_2_).

## LIST OF ABBREVIATIONS

4-HNE: 4-hydroxynonenal
4α-PDD: 4α-phorbol-12,13-decanoate
ASIC: Acid-sensing Ion Channel
AITC: Allyl Isothiocyanate
αβmeATP: AlphabetameATP
AA: Arachidonic Acid
CAMKII: Calcium Calmodulin-dependent Protein Kinase II
CFA: Complete Freund’s Adjuvant
CNO: Clozapine-N-oxide
CPP: Conditioned Place Preference
CRF2: Corticotropin Releasing Factor 2
DOMS: Delayed-onset Muscle Soreness
DPHG: Dihydroxyphenylglycine
DRG: Dorsal Root Ganglion
EET: Epoxyeicosatrienoic Acid
EHS: Exertional Heat Stroke
GDNF: Glial Cell-derived Neurotrophic Factor
GFP: Green Fluorescent Protein
HSV: Herpes Simplex Virus
H_2_O_2_: Hydrogen Peroxide
IHC: Immunohistochemistry
IASP: International Association for the Study of Pain
ICD-11: International Classification of Diseases 11
IB4: Isolectin B4
LC: Lengthening Contraction
MH: Malignant Hyperthermia
MAPK: Mitogen-activated Protein Kinase
MGS: Mouse Grimace Scale
NGF: Nerve Growth Factor
NMDA: N-methyl-D-aspartate
NMDAR: N-methyl-D-aspartate Receptor
NSAIDs: Non-steroidal Anti-inflammatory Drugs
Nrf2: Nuclear Factor Erythroid 2-related Factor 2
NF-κB: Nuclear Factor-kappa B
OLAMs: Oxidized Linoleic Acid Metabolites
PMA: Phorbol 12-myristate 13-acetate
pERK: Phosphor Extracellular Signal-regulated Kinase
PRICE: Protection, Rest, Ice, Compression, and Elevation
PKA: Protein Kinase A
PKC: Protein Kinase C
RGS: Rats Grimace Scale
RT-PCR: Real Time Reverse Transcriptase-polymerase Chain Reaction
RT-qPCR: Real Time Reverse Transcriptase-polymerase Chain Reaction
RTX: Resiniferatoxin
SR: Sarcoplasmic Reticulum
TMD: Temporomandibular Disorders
TRP: Transient Receptor Potential
TRPA: Transient Receptor Potential Cation Channel Subfamily Ankyrin
TRPA1: Transient Receptor Potential Cation Channel Subfamily Ankyrin Member 1
TRPC: Transient Receptor Potential Cation Channel Subfamily Canonical
TRPC1: Transient Receptor Potential Cation Channel Subfamily Canonical Member 1
TRPM: Transient Receptor Potential Cation Channel Subfamily Melastatin
TRPM8: Transient Receptor Potential Cation Channel Subfamily Melastatin Member 8
TRPML: Transient Receptor Potential Cation Channel Subfamily Mucolipin
TRPP: Transient Receptor Potential Cation Channel Subfamily Polycystin
TRPV: Transient Receptor Potential Cation Channel Subfamily Vanilloid
TRPV1: Transient Receptor Potential Cation Channel Subfamily Vanilloid Member 1
TRPV2: Transient Receptor Potential Cation Channel Subfamily Vanilloid Member 2
TRPV4: Transient Receptor Potential Cation Channel Subfamily Vanilloid Member 4
TG: Trigeminal Ganglion
Vc: Trigeminal Spinal Subnucleus Caudalis
WB: Western Blot
WHO: World Health Organization
